# Supplementary material for: The Effect of Feeding with Central European Local Mulberry Genotypes on the Development and Health Status of Silkworms and Quality Parameters of Raw Silk
Source: Insects. 2022 Sep 14;13(9):836. doi: 10.3390/insects13090836 (PMC9506520; doi:10.3390/insects13090836)
Supplement: Supplementary file 1 [file insects-13-00836-s001.zip › insects-1915282-re-supplementary.pdf]

Table S1. List of local Slovenian and Hungarian mulberry genotypes planted in the mulberry collection with location, geographic coordinates and detailed data, along with the list and specification of reference sericultural and fruit varieties.

Identification No.	region	regionalization	Abb.	location	samp. date	Lat. (° N)	Long. (° E)	circ. (cm)
Slovenian genotypes								
SE 5	South-Eastern	Bela krajina	SE	Breznik	6.7.2016	45.520700	15.159010	122
SE 8	South-Eastern	Bela krajina	SE	Marindol	6.7.2016	45.506450	15.329100	94
SM 6	Submediterranean	Ajdovščina	SM	Črniče	11.2.2016	45.904970	13.780760	250
SM 101.1	Submediterranean r	Kras	SM	Famlje	22.2.2016	45.660898	14.006956	189
SM 137	Submediterranean	Koprska brda	SM	Sečovlje	22.2.2016	45.465650	13.646830	237
SM 214	Submediterranean	Goriška brda	SM	Medana	4.7.2016	45.982020	13.519870	120
SP 12	Subpannonean	Slovenske gorice	SP	Benedikt	20.7.2016	46.614460	15.887180	281
SP 256	Subpannonean	Murska ravan	SP	Dobrovnik	26.7.2016	46.655350	16.354410	178
SP 303	Subpannonean	Dravska ravan	SP	Prepolje	8.6.2017	46.445965	15.764567	340
SP 304	Subpannonean	Dravska ravan	SP	Župečja vas	8.6.2017	46.375654	15.766740	267
Hungarian genotypes								
BA 2151	Southern Transdanubia	Baranya	BA	Bogadmindszent	14.6.2017	45.916110	18.054140	132
BA 2225	Southern Transdanubia	Baranya	BA	Pecsvarad	15.6.2017	46.134550	18.377724	290
BE 1264.2	Southern Great Plain	Bekes	BE	Bekescsaba	5.7.2017	46.684300	21.087600	182
GMS 2286	Western Transdanubia	Gyor-Moson-Sopron	GMS	Bosarkany	20.6.2017	47.713730	17.223230	330
GMS 2329	Western Transdanubia	Gyor-Moson-Sopron	GMS	Lebeny	20.6.2017	47.737740	17.386580	270
GMS 2532	Western Transdanubia	Gyor-Moson-Sopron	GMS	Nagycenk	20.6.2017	47.610360	16.672090	245
GMS 2533	Western Transdanubia	Gyor-Moson-Sopron	GMS	Nagycenk	20.6.2017	47.610360	16.672090	225
SO 1013	Southern Transdanubia	Somogy	SO	Nagybajom	30.5.2017	46.395783	17.499668	2x70
SO 1035	Southern Transdanubia	Somogy	SO	Mernye	30.5.2017	46.516639	17.819101	2x65
SO 1042	Southern Transdanubia	Somogy	SO	Igal	30.5.2017	46.533231	17.939263	340
TO 1131	Southern Transdanubia	Tolna	TO	Sárszentlőrinc	14.6.2017	46.664900	18.598900	483
VE 2706	Central Transdanubia	Veszprem	VE	Tihany	27.6.2017	46.922300	17.824900	260
ZA 1060	Western Transdanubia	Vas	VA	Bajánsenye	6.6.2017	46.807400	16.390300	172

ZA 1070	Western Transdanubia	Vas	VA	Csákánydoroszló	6.6.2017	46.967800	16.470300	272
ZA 2084	Western Transdanubia	Zala	ZA	Kiliman - Gelse	6.6.2017	46.628430	16.995460	60

Reference sericultural varieties	origin	obtained	prop./planting date
Morus alba 'Florio'	Italy	mulberry gene bank CREA Padua, Italy	3.2015
Morus alba 'Giazzola'	Italy	mulberry gene bank CREA Padua, Italy	3.2015/
Morus alba 'Kokusou'	Japan	mulberry gene bank CREA Padua, Italy	3.2015
Morus alba 'Morettiana'	Italy	mulberry gene bank CREA Padua, Italy	3.2015
Fruit varieties			
M. alba s.l. (Bulgaria)	Bulgaria	Hubmann, Austria (original from collector in Bulgaria)	2014/3.2016
M. alba × *rubra*	hybrid	Hubmann, Austria (original Graz, Leonhardstr.)	2014/3.2016
M. australis	unknown	Hubmann, Austria (original from cornusmas.eu)	2014/3.2016
M. nigra	East Styria, Austria	Pucher, Austria	2014/3.2016

Table S2. The mean concentrations of the total proteins, total phenolics and individual phenolics (mg/g DW) in leaves of Slovenian, Hungarian old mulberry genotypes, reference sericultural and fruit varieties. Among predominant phenolics, different letters (a–n) indicate significant differences (p < 0.05), which were determined using the post hoc Duncan test.

Genotype name	tProt.	tPH	chlorogenic a.	4–caffeoyl–QA	5–caffeoyl–QA	total caffeoyl–QA	c–5–CQA	t–5–CQA	p–CAH	p–CAH 2	p–CQA	tCQA	rutin	Q–3–glu	QMH	Q–diR–gly	QRH	Q–ac-etyl–RH	QAH	t Q–gly	KAH	K–diRH	KRH	K–ace-tyl–RH	t K gly. *
Slovenian genotypes																									
SE 5	222.14	19.35	11.73 ef	0.89 b–e	0.23 ab	12.84	1.09	0.92	0.24	0.78	0.08	3.11	3.16 b–d	0.34 d–g	1.64	0.035	0.013	0.005	0.076	5.27	1.72 de	0.16	0.62	0.03	2.53
SE 8	217.93	16.71	9.66 f–i	0.73 d–g	0.23 ab	10.62	1.71	0.97	0.23	1.81	0.20	4.92	1.94 j–l	0.24 j–m	1.57	0.051	0.014	0.002	0.077	3.89	2.16 g–k	0.28	0.39	0.06	2.89
SM 6	182.65	11.26	5.10 f–i	0.41 b–e	0.15 ab	5.66	0.45	0.31	0.19	0.74	0.09	1.78	2.62 b–e	0.33 d–g	1.01	0.063	0.013	0.002	0.043	4.08	1.08 r	0.21	0.45	0.05	1.80
SM 101.1	239.42	17.54	8.44 g–i	1.06 b–e	0.22 ab	9.73	1.60	1.48	0.52	0.81	0.26	4.67	2.94 b–e	0.29 f–j	1.93	0.134	0.028	0.004	0.094	5.42	2.04 h–l	0.20	0.48	0.11	2.84
SM 137	211.04	15.97	7.76 hi	0.74 d–g	0.23 ab	8.73	1.45	0.87	0.44	1.55	0.20	4.51	2.85 b–f	0.37 d	1.31	0.131	0.050	0.003	0.068	4.78	2.17 g–j	0.20	0.82	0.14	3.33
SM 214	193.00	16.22	7.49 i	1.18 b–d	0.25 ab	8.91	1.30	0.94	0.76	1.48	0.22	4.70	1.34 mn	0.26 i–l	1.01	0.090	0.024	0.001	0.056	2.78	1.45 o–r	0.14	0.28	0.13	1.99
SP 12	188.59	11.80	9.15 f–i	0.24 g	0.04 c	9.43	1.89	0.06	0.82	1.47	0.29	2.25	2.03 h–l	0.18 mn	1.51	0.022	0.012	0.001	0.072	3.83	3.22 bc	0.22	0.56	0.02	4.03
SP 256	186.99	18.53	8.79 f–i	0.79 c–f	0.20 ab	9.78	0.91	0.60	0.20	1.02	0.05	2.77	1.97 i–l	0.20 mn	1.66	0.033	0.012	0.001	0.079	3.95	2.26 f–j	0.16	0.45	0.05	2.91
SP 303	229.61	17.41	10.42 e–i	0.89 b–e	0.23 ab	11.53	1.84	1.17	0.35	1.87	0.26	5.49	1.86 jm	0.28 h–k	1.56	0.066	0.021	0.000	0.073	3.85	2.70 de	0.43	0.49	0.09	3.71
SP 304	214.57	16.82	8.69 f–i	1.02 b–e	0.27 ab	9.98	1.67	0.92	0.90	1.44	0.19	5.12	2.30 g–j	0.22 k–n	1.37	0.018	0.016	0.007	0.062	4.00	2.34 e–h	0.10	0.56	0.05	3.04
Hungarian genotypes																									
BA 2151	202.78	16.75	7.12 f–i	1.07 b–e	0.36 ab	8.55	0.76	0.07	0.46	0.49	0.06	1.84	1.87 j–l	0.21 l–n	0.91	0.084	0.017	0.001	0.041	3.13	1.02 r	0.03	0.34	0.09	1.46
BA 2225	209.72	17.08	5.93 f–i	1.22 b–e	0.25 ab	7.41	0.93	0.27	0.38	0.65	0.06	2.29	1.25 mn	0.05 n	0.74	0.024	0.007	0.000	0.031	2.10	0.94 r	0.06	0.22	0.04	1.25
BE 1264.2	225.90	17.84	10.48 d–i	0.94 b–e	0.25 ab	11.66	1.80	1.38	0.58	1.96	0.39	6.10	3.10 b–d	0.29 g–j	2.08	0.023	0.010	0.000	0.107	5.61	3.17 bc	0.24	0.76	0.05	4.22
GMS 2286	215.79	17.58	9.09 f–i	1.05 b–e	0.28 ab	10.42	1.58	0.93	0.53	2.09	0.16	5.28	2.93 b–e	0.35 de	1.48	0.086	0.022	0.004	0.073	4.94	1.89 j–n	0.05	0.66	0.07	2.66
GMS 2329	219.85	17.16	11.09 d–g	0.92 b–e	0.23 ab	12.24	1.96	0.98	0.47	1.69	0.13	5.23	2.31 e–i	0.30 e–i	1.78	0.020	0.010	0.004	0.479	4.89	1.93 i–l	0.38	0.47	0.04	2.82
GMS 2532	210.49	18.81	10.00 f–i	1.26 bc	0.21ab	11.46	1.06	0.31	0.57	0.62	0.08	2.63	3.06 b–d	0.19 mn	0.99	0.024	0.014	0.002	0.048	4.32	1.56 m–r	0.46	0.75	0.03	2.80
GMS 2533	178.81	17.88	9.17 f–i	1.22 b–d	0.21 ab	10.60	0.97	0.27	0.64	0.73	0.09	2.70	2.68 c–g	0.17 n	0.87	0.023	0.012	0.002	0.041	3.79	1.33 r	0.47	0.65	0.03	2.48
SO 1013	221.89	16.95	9.63 f–i	0.92 b–e	0.24ab	10.79	1.80	0.67	0.44	2.04	0.15	5.10	2.38 f–j	0.22 l–n	1.26	0.057	0.015	0.002	0.061	4.00	1.78 k–o	0.06	0.57	0.06	2.48
SO 1035	220.60	18.62	13.52 cd	0.79 c–f	0.26 ab	14.56	2.33	1.20	0.42	1.46	0.36	5.77	2.31 g–j	0.31 d–i	1.96	0.080	0.027	0.003	0.093	4.78	3.26 b	0.31	0.62	0.13	4.32
SO 1042	213.87	18.21	4.00 f–i	0.61 b–e	0.26 ab	4.87	0.56	0.29	0.23	0.77	0.04	1.89	1.96 j–l	0.20 l–n	1.18	0.019	0.010	0.000	0.056	3.42	1.30 r	0.05	0.34	0.03	1.72
TO 1013	214.70	19.21	7.79 f–i	1.35 f–g	0.27 c	9.40	0.82	0.16	0.55	0.65	0.08	2.26	3.35 a	0.21 de	0.74	0.041	0.013	0.002	0.041	4.40	0.99 h–l	0.21	0.65	0.03	1.87
TO 1131	212.70	18.87	10.66 d–h	1.37 b	0.24 ab	12.27	2.00	0.57	0.74	1.78	0.40	5.49	4.31 a	0.37 d	1.39	0.026	0.008	0.003	0.078	6.18	2.27 f–i	0.50	1.22	0.03	4.02
VE 2706	196.71	19.04	8.80 f–i	0.66 e–g	0.24 ab	9.71	0.87	0.91	0.49	0.92	0.18	3.37	2.70 b–g	0.36 d	2.05	0.092	0.034	0.003	0.103	5.34	2.87 cd	0.03	0.71	0.08	3.69
ZA 1060	226.89	19.33	11.27 d–g	0.64 e–g	0.22 ab	12.12	1.08	0.77	0.15	1.13	0.21	3.34	2.48 e–i	0.45 c	2.03	0.119	0.035	0.001	0.092	5.21	2.71 de	0.04	0.46	0.21	3.42

ZA 1070	210.18	21.03	10.48 d–i	0.98 b–e	0.19 b	11.64	1.08	0.64	0.29	1.10	0.07	3.17	3.18 bc	0.32 d–h	1.88	0.037	0.014	0.004	0.092	5.53	1.53 n–r	0.43	0.47	0.02	2.45
ZA 2084	202.88	18.11	7.80 hi	0.92 b–e	0.22 ab	8.94	1.19	1.00	0.56	1.61	0.16	4.51	1.91 j–l	0.26 i–l	1.56	0.066	0.022	0.003	0.084	3.90	2.62 df	0.22	0.44	0.09	3.37
Ref. var.																									
Florio'	197.92	20.54	13.27 c–e	1.23 bd	0.23 ab	14.73	1.13	0.53	0.54	1.14	0.12	3.47	3.10 b–d	0.29 g–j	1.83	0.040	0.015	0.002	0.082	5.36	2.51 d–g	0.60	0.70	0.05	3.86
Giazzola'	178.72	19.15	8.49 g–i	1.08 b–e	0.21 ab	9.77	0.90	0.28	0.56	0.65	0.10	2.49	2.64 dh	0.24 j–n	0.88	0.441	0.020	0.004	0.041	4.26	1.28 r	0.15	0.60	0.07	2.10
Kokusou'	205.92	21.36	17.67 ab	0.89 b–e	0.21 ab	18.77	1.68	0.77	0.19	1.05	0.14	3.82	4.10 a	0.54 b	2.25	0.023	0.008	0.001	0.097	7.02	3.20 bc	0.10	1.07	0.02	4.38
Morettiana'	182.94	18.75	7.44 i	0.93 b–e	0.21 ab	8.58	0.78	0.67	0.75	1.33	0.08	3.61	3.22 b	0.52 b	1.56	0.250	0.039	0.001	0.089	5.68	1.35 pr	0.33	0.45	0.10	2.23
Fruit var.																									
M. alba (Bulgaria)	151.50	19.62	10.60 d–h	0.99 b–e	0.19 ab	11.78	1.16	0.22	0.23	0.38	0.41	2.40	1.07 n	0.17 n	1.00	0.046	0.016	0.001	0.046	2.35	1.97 h–l	0.43	0.36	0.10	2.86
M. alba × *rubra*	203.84	21.51	14.90 bc	4.36 a	0.28 a	19.54	0.78	2.41	2.03	1.76	0.41	7.39	1.67 km	0.74 a	1.54	0.161	0.082	0.001	0.088	4.28	4.42 a	0.48	0.79	0.59	6.27
M. australis	179.51	17.48	8.71 f–i	1.37 b	0.20 ab	10.27	0.93	0.42	0.92	1.02	0.20	3.49	1.58 ln	0.20 mn	1.08	0.031	0.018	0.001	0.069	2.98	1.73 l–p	0.46	0.34	0.08	2.60
M. nigra	200.93	20.26	18.05 a	1.26 bc	0.24 ab	19.55	3.24	1.99	1.15	2.35	1.27	10.00	2.18 g–k	0.35 df	1.30	0.057	0.018	0.002	0.054	3.96	1.27 r	0.21	0.34	0.03	1.84

*Ref. var., reference sericultural varieties; Fruit var, fruit varieties; tProt, total protein; tPH, total phenolics; 4-caffeoyl-QA, 4-caffeoylquinic acid; 5-caffeoyl-QA, 5-caffeoylquinic acid; 5-total CQA, total caffeoylquinic acid derivatives; c-5-CQA, c-5-coumaroylquinic acid; t-5-CQA, t-5-coumaroylquinic acid; p-CAH, p-coumaric acid hexoside; p-CAH2, p-coumaric acid hexoside 2; p-CQA, p-coumaroylquinic acid; total CQA, total coumaroylquinic acid derivatives; Q-3-glu, quercetin-3-glucoside; QMH, quercetin malonyl-hexoside; Q-diR-gly, quercetin dirhamnosyl-glycoside; QRH, quercetin rhamnosyl-hexoside; Q-acetyl-RH, quercetin acetyl-rhamnosyl hexoside; QAH, quercetin acetyl hexoside; total Q-gly, total quercetin glycoside derivatives; KAH, kaemph acetyl-hexoside; K-diRH, kaempheroldirhamnosyl-hexoside; KRH, kaempherol rhamnosyl-hexoside; K-acetyl-RH, kaempherol acetyl-rhamnosyl-hexoside; t K gly, total kaempherol-glycoside derivatives

Table S3. The mean concentrations of the macro- and micronutrients in leaves of Slovenian, and Hungarian old mulberry genotypes, reference sericultural and fruit varieties.

Genotype name	P	S	K	Ca	Cl	Mn	Fe	Ni	Zn	Rb	Sr
Slovenian mulberry genotypes											
SE 5	2.04	0.79	16.20	14.40	1110	57.8	141	21.5	18.5	16.9	54
SE 8	1.79	0.91	17.30	16.90	922	64.5	136	17.1	23.8	19.5	61.1
SM 6	1.54	0.60	12.60	17.90	774	71.5	149	27.1	33.7	31.7	72
SM 101.1	1.47	0.94	13.80	11.60	543	70	136	18.7	16.9	51.7	54.9
SM 137	1.61	0.76	16.90	19.80	1040	73.8	146	35.6	20.9	15.8	87.4
SM 214	1.57	1.13	15.50	16.50	897	49	127	19	11.4	29.6	37.8
SP 12	1.32	0.86	13.60	13.20	665	64.9	120	15.8	18.4	13.8	57.1
SP 256	1.85	0.91	18.10	16.30	1040	85.5	141	24.7	23.8	46.7	71.7
SP 303	1.41	0.97	14.70	10.80	850	59.6	156	18.8	26.3	28	42.8
SP 304	1.25	1.04	14.30	19.50	872	83.1	133	20.7	14	27.6	71.8
Hungarian mulberry genotypes											
BA 2151	1.40	0.88	20.12	10.32	765	95	170	23	30	35	74.9
BA 2225	1.50	0.89	22.10	11.20	818	105	172	24.9	31.5	39.1	75.9
BE 1264.2	1.65	0.98	18.40	14.10	1320	62	115	26.3	25.5	23	51.4
GMS 2286	1.56	0.93	18.40	12.90	754	79	155	27.6	18.4	45.9	57.9
GMS 2329	1.35	0.79	15.60	12.30	838	82.1	145	19.5	20.8	36.9	80.6
GMS 2532	1.99	1.26	18.60	20.00	994	101	185	24.9	28	31.6	70.9
GMS 2533	1.51	1.09	13.50	19.20	840	97.2	204	35.5	19.1	22.7	152
SO 1013	1.59	0.77	16.40	14.60	925	51.4	101	19	17.5	12.7	46
SO 1035	1.46	0.66	17.80	8.63	609	56	132	17.8	16.9	27.3	22.8
SO 1042	1.54	0.92	14.50	13.40	820	89.5	178	21.2	22.4	36.4	80.4
TO 1013	1.59	0.77	16.40	14.60	925	51.4	101	19	17.5	12.7	46
TO 1131	1.61	0.80	16.90	9.41	660	47.9	106	13.5	21.1	23.8	34.4
VE 2706	1.64	0.86	14.80	15.70	1330	80.2	140	14.5	13.6	25.5	70.8
ZA 1060	1.64	1.04	16.50	11.30	1000	61	144	14.6	25.2	17	41.8
ZA 1070	1.76	0.94	15.90	13.60	816	71	142	17.9	21.6	35.1	66.6
ZA 2084	1.43	0.96	15.80	12.90	994	73.8	142	27.3	24	42.1	57.9
Reference sericultural varieties											
Florio'	1.72	0.77	21.40	14.40	535	61.1	132	33.4	15.7	31.9	30.6
Giazzola'	1.33	0.78	11.20	16.10	1010	49.5	110	17.5	16.4	19.9	44.8
Kokusou'	1.71	0.92	14.30	11.10	460	50.1	114	20.3	26.1	59.4	36.1
Morettiana'	1.32	0.91	17.70	16.90	936	47.4	157	15.5	14.6	13.6	28.9
Fruit varieties											
M. alba (Bulgaria)	1.35	0.68	19.20	11.50	511	44.2	121	28.7	19.8	21.8	53.7
M. alba × *rubra*	1.29	0.60	16.00	12.60	339	40	120	20	20.6	33.4	41.3
M. australis	1.50	0.74	18.40	17.60	668	49.6	110	20.4	21.6	15.8	57.5
M. nigra	1.07	0.46	16.70	15.10	759	47.7	127	25.5	11.6	18.6	77.6

Table S4. Differences in mean silkworm weight, fresh cocoon weight and silk thread parameters among Slovenian, Hungarian mulberry varieties, reference sericultural varieties and fruit varieties.

Group name	mean silkworm weight 5th–7th day 5th instar)	Cocoon FW*	silk thread parameters				reeling wastes	
			length<br>m ± sd	weight<br>g ± sd	thickness<br>denier ± sd	breaks median,<br>min, max	struse<br>g ± sdg	telette<br>g ± sd
Slovenian mulberry genotypes								
SE 5	4.34 ± 0.42 a–c	2.2	1411.6 ± 119.8 b–h	0.449 ± 0.035 a–e	2.876 ± 0.259 a–e	0, 0, 1	0.027 ± 0.010 c–d	0.018 ± 0.004 a–e
SE 8	4.61 ± 0.37 a–c	2.27	1417.2 ± 153.1 b–h	0.453 ± 0.045 a–e	2.911 ± 0.446 a–d	0, 0, 0	0.035 ± 0.013 b–d	0.014 ± 0.006 b–e
SM 6	3.87 ± 0.36 a–c	1.97	1324.0 ± 180.6 d–i	0.375 ± 0.031 f–i	2.587 ± 0.379 c–e	0, 0, 0	0.040 ± 0.015 b–d	0.010 ± 0.004 de
SM 101.1	3.83 ± 0.57 bc	2.07	1439.8 ± 107.4 b–f	0.431 ± 0.034 a–g	2.702 ± 0.219 b–e	0, 0, 1	0.033 ± 0.009 c–d	0.013 ± 0.003 b–e
SM 137	3.82 ± 0.37 bc	2.11	1441.2 ± 80.2 b–f	0.407 ± 0.037 c–h	2.546 ± 0.269 de	0, 0, 0	0.038 ± 0.011 b–d	0.017 ± 0.005 a–e
SM 214	4.78 ± 0.43 a–c	2.19	1424.3 ± 184.7 b–g	0.424 ± 0.042 b–g	2.690 ± 0.164 b–e	0, 0, 0	0.034 ± 0.012 b–d	0.018 ± 0.009 a–e
SP 12	4.97 ± 0.44 a–c	2.35	1455.3 ± 143.7 b–f	0.457 ± 0.031 a–e	2.849 ± 0.300 a–e	0, 0, 0	0.043 ± 0.014 a–d	0.029 ± 0.014 a–c
SP 256	4.42 ± 0.42 a–c	2.08	1532.3 ± 174.8 ab	0.438 ± 0.054 a–f	2.577 ± 0.162 c–e	0 ,0 ,1	0.036 ± 0.013 b–d	0.013 ± 0.009 b–e
SP 303	4.17 ± 0.27 a–c	2.01	1437.9 ± 142.6 b–f	0.431 ± 0.028 a–g	2.711 ± 0.171 b–e	0, 0, 0	0.039 ± 0.013 b–d	0.018 ± 0.012 a–e
SP 304	4.57 ± 0.50 a–c	2.1	1307.3 ± 109.6 d–i	0.405 ± 0.029 c–h	2.800 ± 0.256 a–e	0, 0, 0	0.039 ± 0.012 b–d	0.016 ± 0.004 b–e
Hungarian mulberry genotypes								
BA 2151	5.17 ± 0.46 a	2.38	1404.0 ± 160.3 b–h	0.430 ± 0.051 a–g	2.776 ± 0.351 a–e	0, 0, 2	0.044 ± 0.019 a–d	0.021 ± 0.012 a–e
BA 2225	4.13 ± 0.38 a–c	2.1	1531.1 ± 228.2 b–i	0.487 ± 0.042 a–g	2.918 ± 0.495 a–e	0 ,0 ,2	0.019 ± 0.010 a–d	0.023 ± 0.020 a–e
BE 1264.2	4.48 ± 0.35 a–c	2.26	1367.2 ± 189.9a b–i	0.471 ± 0.040 a–c	3.143 ± 0.428 a	0, 0, 0	0.026 ± 0.015 c–d	0.018 ± 0.006 a–e
GMS 2286	4.53 ± 0.42 a–c	2.23	1627.0 ± 164.9 ab	0.468 ± 0.052 a–d	2.601 ± 0.261 b–e	0 ,0 ,1	0.043 ± 0.014 a–d	0.015 ± 0.010 a–e
GMS 2329	4.73 ± 0.28 a–c	2.38	1315.5 ± 106.6 d–i	0.433 ± 0.044 a–g	2.966 ± 0.264 a–c	0, 0, 1	0.054 ± 0.015 a–c	0.016 ± 0.010 b–e
GMS 2532	4.91 ± 0.46 a–c	2.29	1390.3 ± 112.2 b–i	0.433 ± 0.052 a–g	2.806 ± 0.294 a–e	0, 0, 1	0.054 ± 0.021 a–c	0.027 ± 0.016 a–d
GMS 2533	4.54 ± 0.24 a–c	2.19	1331.3 ± 305.4 b–i	0.420 ± 0.102 c–g	2.831 ± 0.356 a–e	0, 0, 5	0.048 ± 0.007 a–d	0.057 ± 0.076 a
SO 1013	5.11 ± 0.27 ab	2.45	1499.1 ± 174.9 a–d	0.494 ± 0.160 a	2.973 ± 0.878 a–c	0, 0, 2	0.046 ± 0.020 a–d	0.014 ± 0.004 a–e
SO 1035	4.21 ± 0.24 a–c	2.23	1520.6 ± 262.6 a–c	0.462 ± 0.066 a–d	2.782 ± 0.422 a–e	0 ,0 ,0	0.032 ± 0.024 c–d	0.017 ± 0.006 a–e
SO 1042	4.54 ± 0.37 a–c	2.18	1412.8 ± 131.9 b–h	0.432 ± 0.032 a–g	2.758 ± 0.130 a–e	0, 0, 0	0.045 ± 0.013 a–d	0.013 ± 0.006 c–e

TO 1131	5.11 ± 0.27 ab	2.45	1495.8 ± 139.6 a–d	0.446 ± 0.038 a–b	2.699 ± 0.294 a–d	0, 0, 3	0.047 ± 0.020 b–d	0.017 ± 0.005 a–e
VE 2706	4.33 ± 0.31 a–c	2.2	1277.0 ± 166.6 f–i	0.356 ± 0.063 hi	2.554 ± 0.597 c–e	0, 0, 7	0.072 ± 0.018 a–c	0.015 ± 0.005 b–e
ZA 1060	3.74 ± 0.29 c	2.18	1393.8 ± 202.4 b–h	0.428 ± 0.046 a–g	2.793 ± 0.343 a–e	0, 0, 2	0.042 ± 0.016 a–d	0.022 ± 0.008 a–e
ZA 1070	5.05 ± 0.29 ab	2.3	1384.1 ± 143.5 d–i	0.422 ± 0.040 d–h	2.766 ± 0.313 a–e	0, 0, 7	0.057 ± 0.027 a–c	0.027 ± 0.021 a–e
ZA 2084	4.44 ± 0.46 a–c	2.31	1383.3 ± 146.6 a–d	0.420 ± 0.037 a–d	2.740 ± 0.156 a–e	0, 0, 1	0.038 ± 0.011 c–d	0.024 ± 0.018 a–e
Reference sericultural varieties								
'Florio'	5.08 ± 0.23 ab	2.34	1312.8 ± 131.3 d–i	0.439 ± 0.036 a–f	3.019 ± 0.226 ab	0, 0, 1	0.051 ± 0.024 a–d	0.013 ± 0.009 c–e
'Giazzola'	4.56 ± 0.23 a–c	2.2	1267.5 ± 212.9 f–j	0.402 ± 0.063 d–h	2.866 ± 0.231 a–e	0, 0, 7	0.045 ± 0.012 a–d	0.020 ± 0.021 a–e
'Kokusou'	4.75 ± 0.84 a–c	2.2	1401.5 ± 200.2 b–h	0.429 ± 0.025 a–g	2.793 ± 0.314 a–e	0, 0, 1	0.039 ± 0.010 b–d	0.021 ± 0.011 a–e
'Morettiana'	4.71 ± 0.38 a–c	2.17	1335.0 ± 168.3 c–i	0.411 ± 0.028 c–h	2.803 ± 0.347 a–e	0, 0, 1	0.042 ± 0.017 a–d	0.022 ± 0.021 a–e
Fruit varieties								
M. alba (Bulgaria)	4.69 ± 0.39 a–c	2.15	1201.8 ± 152.3 ij	0.343 ± 0.110 ij	2.634 ± 0.885 b–e	0, 0, 0	0.045 ± 0.016 a–d	0.019 ± 0.012 a–e
M. alba × rubra	4.19 ± 0.58 a–c	2.09	1242.8 ± 229.8 g–j	0.370 ± 0.078 g–i	2.674 ± 0.203 b–e	0, 0, 1	0.053 ± 0.016 a–c	0.030 ± 0.022 ab
M. australis	4.87 ± 0.19 a–c	2.35	1289.8 ± 142.7 e–i	0.409 ± 0.037 c–h	2.871 ± 0.279 a–e	0, 0, 1	0.022 ± 0.011 d	0.017 ± 0.009 a–e
M. nigra	4.93 ± 0.52 a–c	n.d.	–	–	–	–	–	–

*FW, fresh weight

Table S5. PCA scores of the main PC1 and PC2 axes.

	**PC 1**	**PC 2**
5th C/5th D larval weight	0.38958	0.26108
5th C/6th D larval weight	0.6744	0.077085
5th C/7th D larval weight	0.53864	0.040782
cocoon FW	0.29208	0.46757
cocoon DW	0.36755	0.48416
raw silk length	–0.60646	0.20252
raw silk weight	–0.2226	0.33477
raw silk thickness	0.45623	0.22308
total proteins	–0.62196	0.23758
total phenolics	0.79583	0.32608
chlorogenic acid	0.5859	0.005993
4–caffeoquinic acid	0.40289	0.24664
c–5–coumaroylquinnic a.	–0.30679	0.045554
rutin	0.69188	0.07261
quercetin–3–glucoside	0.66188	–0.12959
quercetin malonyl–hexoside	0.21189	0.028206
kaempherol acetyl–hexoside	–0.0156	–0.02001
P	–0.1901	0.049048
S	–0.24099	0.1504
Cl	–0.39781	0.1778
K	0.088651	0.29482
Ca	–0.06974	–0.33728
Mn	–0.62261	0.36901
Fe	–0.30873	0.25593
Ni	–0.01639	–0.00892
Zn	–0.30134	0.16575
Rb	0.11663	0.050663
Sr	–0.60829	0.21218
Eigenvalue	4.74754	1.32782
% variance	78.144	21.856
